# Targeting the MAPK Pathway in Brain Tumors: Mechanisms and Therapeutic Opportunities

**DOI:** 10.3390/cancers18010156

**Published:** 2026-01-02

**Authors:** Dimitrios Vrachas, Elisavet Kosma, Angeliki-Ioanna Giannopoulou, Angeliki Margoni, Antonios N. Gargalionis, Elias A. El-Habr, Christina Piperi, Christos Adamopoulos

**Affiliations:** 1 Department of Biological Chemistry, Medical School, National and Kapodistrian University of Athens, 11527 Athens, Greece; dimitrisvra@med.uoa.gr (D.V.); elisavet3kosma@gmail.com (E.K.); angelig@med.uoa.gr (A.-I.G.); angeliki.margoni@gmail.com (A.M.); elias.el-habr@sorbonne-universite.fr (E.A.E.-H.); cpiperi@med.uoa.gr (C.P.); 2 Laboratory of Clinical Biochemistry, Medical School, ‘Attikon’ University General Hospital, National and Kapodistrian University of Athens, 12462 Athens, Greece; agargal@med.uoa.gr; 3 Sorbonne Université, CNRS, INSERM, Institut de Biologie Paris Seine, Center for Neuroscience at Sorbonne Université, 75005 Paris, France; 4 Department of Oncological Sciences, Icahn School of Medicine at Mount Sinai, New York, NY 10029, USA

**Keywords:** MAPK signaling, brain tumors, RAF-MEK-ERK pathway, *BRAF V600E* mutation, *BRAF-KIAA1549* fusion, targeted therapy, RAF inhibitors, MEK inhibitors

## Abstract

Central nervous system (CNS) tumors consist of a diverse set of malignancies that remain clinically challenging due to their biological complexity, high morbidity, and limited responsiveness to current therapies. A growing body of genomic evidence has revealed that dysregulation of the mitogen-activated protein kinase (MAPK) signaling pathway is a recurrent and unifying characteristic across many pediatric and adult CNS tumor entities. Alterations affecting upstream receptor tyrosine kinases (RTKs), RAS GTPases, RAF kinases, and other associated regulators contribute to MAPK signaling pathway hyperactivation, shaping tumor behavior, therapy response and resistance. These aberrations ranging from hotspot mutations such as *BRAF V600E* and oncogenic fusions like *BRAF–KIAA1549* are particularly enriched in gliomas and glioneuronal tumors, highlighting MAPK signaling as a key oncogenic driver. The expanding availability of molecularly targeted compounds, including selective inhibitors of RAF, MEK and ERK, has begun to transform treatment approaches for specific molecular subtypes. However, the clinical benefit of MAPK-directed therapies is frequently limited by restricted blood–brain barrier (BBB) penetration, intratumoral heterogeneity, parallel pathway reactivation, and an immunosuppressive tumor microenvironment (TME). In this review, we synthesize current knowledge on MAPK pathway alterations in CNS tumors and evaluate the therapeutic landscape of MAPK inhibition, with emphasis on approved agents, emerging compounds, combination strategies, and novel drug-delivery technologies. We also discuss mechanisms that undermine treatment efficacy and highlight future directions aimed at integrating MAPK-targeted therapy into precision-based management of brain tumors.

## Simple Summary

Brain tumors remain among the most difficult cancers to treat, largely because of their biological complexity and the limited ability of many drugs to reach the brain. A major molecular pathway that drives the growth of many brain tumors is the MAPK signaling pathway. In this review, we explain how alterations in this pathway contribute to tumor development in both children and adults, and we summarize current and emerging therapies that specifically target this pathway. We also discuss the main challenges that limit treatment success, including drug resistance, tumor diversity, and the protective blood–brain barrier. By integrating recent advances in molecular biology with therapeutic strategies, this work aims to guide future research and improve precision treatment approaches for patients with brain tumors.

## 1. Introduction

Central nervous system (CNS) tumors represent a heterogeneous group of both malignant and benign entities, characterized by varying clinical behavior, histological, and molecular traits. In 2020, CNS tumors accounted for 1.6% of all cancer cases globally, while in 2022, 321,624 new cases were estimated, corresponding to an age-standardized incidence rate (ASIR) of 3.5 per 100,000 people [1]. Despite their relatively low incidence rate, they impose a major disease burden due to their disproportionately high mortality rates, especially among children [2,3]. The average lifespan for adults with glioblastoma, the most aggressive type of brain tumor, is approximately 2 years [3]. A study utilizing the global burden of disease (GBD) database predicted that the total number of cases will gradually increase by 2040, even though the mortality rates in certain populations may decrease slightly [4]. To date, the current established treatment approaches include surgical resection, radiotherapy, and chemotherapy [5]. CNS tumors are characterized largely by intratumoral heterogeneity which can be defined as the coexistence of genetically, epigenetically, transcriptionally, and phenotypically distinct cell subpopulations within the same tumor mass. This molecular heterogeneity of these tumors, their anatomical location, in conjunction with the protective role of the blood–brain barrier (BBB) and intrinsic or acquired drug resistance, leads to limited treatment efficacy and poor clinical patient outcomes. Importantly, children and young adults who survive by receiving the standard of care experience long-term complications that largely affect their quality of life [3]. Moreover, recent molecular profiling studies have revealed that pediatric and adult gliomas constitute fundamentally distinct biological entities, driven by different oncogenic alterations and signaling dependencies [6,7]. Consequently, there is a pressing need for more targeted and personalized therapeutic strategies.

**Figure 1:**
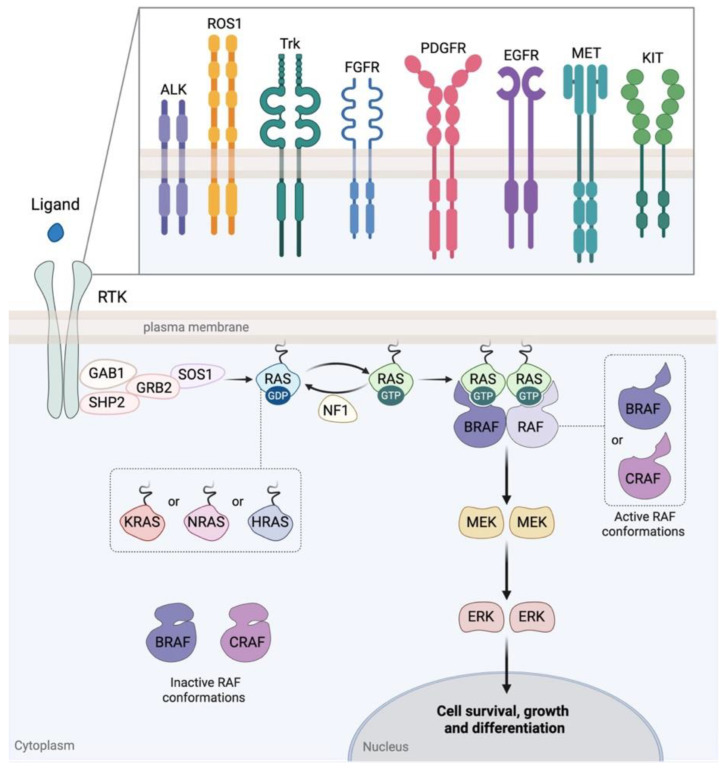
Schematic overview of the MAPK/ERK signaling cascade. Ligand binding to receptor RTKs, including EGFR, FGFR, PDGFR, ALK, ROS1, Trk, MET, and KIT, induces activation of RAS (KRAS, NRAS, or HRAS) through the recruitment of adaptor proteins such as SHP2, GAB1, GRB2, and SOS1. Active GTP-bound RAS, in turn, recruits and activates RAF kinases through dimerization and phosphorylation, forming active BRAF homodimers or BRAF/CRAF heterodimers. RAF then phosphorylates and activates MEK, which consecutively phosphorylates and activates the terminal kinase ERK. Activated ERK translocates to the nucleus, where it phosphorylates transcription factors and co-activators that regulate gene expression. RTK, receptor tyrosine kinase; EGFR, epidermal growth factor receptor; FGFR, fibroblast growth factor receptor; PDGFR, platelet-derived growth factor receptor; ALK, anaplastic lymphoma kinase; ROS1, ROS proto-oncogene 1; Trk, tropomyosin receptor kinase; MET, mesenchymal–epithelial transition factor; KIT, kit proto-oncogene receptor tyrosine kinase; RAS, rat sarcoma viral oncogene; KRAS, Kirsten rat sarcoma viral oncogene homolog; NRAS, neuroblastoma rat sarcoma viral oncogene homolog; HRAS, Harvey rat sarcoma viral oncogene homolog; SHP2, Src homology region 2 domain-containing phosphatase-2; GAB1, GRB2-associated-binding protein 1; GRB2, growth factor receptor-bound protein 2; SOS1, Son of sevenless homolog 1; RAF, rapidly accelerated fibrosarcoma; BRAF, V-Raf murine sarcoma viral oncogene homolog B; CRAF, proto-oncogene c-Raf; MEK, mitogen-activated protein kinase; ERK, extracellular signal-regulated kinase; GTP, guanosine triphosphate; GDP, guanosine diphosphate. Created in BioRender. Adamopoulos, C. (2025) https://BioRender.com/haba8wa (Assessed on 7 September 2025).

The mitogen-activated protein kinase (MAPK) signaling pathway plays a fundamental role in cell physiology by regulating cell cycle, proliferation, survival, differentiation, apoptosis, and is implicated in various other developmental processes [8]. Core components of the MAPK pathway are serine/threonine-specific protein kinases, the mitogen-activated protein kinases (MAPKs), that transduce intracellular signals through sequential protein phosphorylation and activation events. Among them, the rapidly accelerated fibrosarcoma (RAF), the mitogen-activated protein kinase/extracellular signal-regulated kinase (MEK), and the extracellular signal-regulated kinase (ERK) form the RAF-MEK-ERK signaling axis, the central and most thoroughly studied MAPK pathway [8,9] (Figure 1).

The MAPK pathway is usually activated upon binding of a ligand, such as a growth factor, to a receptor tyrosine kinase (RTK), leading to its activation and the following recruitment, intracellularly, of adaptor proteins/regulators, which in turn activate the membrane-anchored small guanosine triphosphatase (GTPase), rat sarcoma virus oncogene (RAS) [10] (Figure 1). Afterward, the active GTP-bound RAS recruits at the membrane and activates RAF through a complex process of dimerization and phosphorylation events [11]. RAF then phosphorylates and activates its substrate MEK, which consecutively phosphorylates and activates ERK [12,13] (Figure 1). Finally, activated ERK phosphorylates its targets, usually transcription factors or co-activators in the nucleus, thereby regulating the expression of several genes [8,14].

RAS-RAF-MEK-ERK signaling axis deregulation, primarily due to its aberrant activation, is a key driver of carcinogenesis [15,16,17,18]. Mutations in key effectors of the pathway, most frequently in *RAS* and *RAF*, have been identified in a wide variety of cancers, including melanomas, lung, colorectal, and ovarian cancers [18,19,20].

Consequently, therapeutic efforts that target MAPK pathway components have led to the approval by the Food and Drug Administration (FDA) of several small-molecule inhibitors, while other alternative targeting approaches are under pre-clinical investigation and development, including gene silencing, proteolysis-targeting chimeras (PROTACs), and bispecific antibodies [8,21,22,23].

Notably, MAPK pathway component alterations have also been detected in primary brain tumors. The most common alterations are the gene fusion between the *BRAF* and *KIAA1549* genes and the *BRAF V600E* mutation, both of which are most prevalent in pediatric compared to adult tumors [24].

Beyond these, diverse genetic alterations involving RTKs, RAS, RAF kinases and pathway regulators lead to the aberrant activation of the MAPK pathway and highlight its central role in the pathogenesis of CNS tumors [8,25].

In this review, we provide a comprehensive overview of MAPK pathway alterations in CNS malignancies, with emphasis on pediatric and adult gliomas, glioneuronal tumors, and ependymomas. We summarize current strategies for MAPK pathway inhibition, including BRAF, MEK, and ERK inhibitors, and discuss how these approaches are being integrated into clinical management [26,27]. Furthermore, we address the major therapeutic challenges that limit efficacy, including restricted BBB penetration, tumor heterogeneity and resistance mechanisms, and the immunosuppressive tumor microenvironment [28,29]. Finally, we highlight emerging treatment concepts and combinatorial approaches that hold promise and shape future perspectives for MAPK-targeted therapy in brain tumors.

## 2. Central Nervous System (CNS) Tumors

Central nervous system (CNS) tumors can be classified as either primary, originating from cell types within the brain and spinal cord, or metastatic, arising from tumors that develop in distal organs, most commonly the lung and breast, and spread to the brain through the bloodstream or the lymphatic system [30,31,32]. Primary CNS neoplasms depict a heterogeneous group, consisting of gliomas, glioneuronal, neuronal, and ependymal tumors [33]. Although relatively rare, tumors of the brain and other parts of the central nervous system contribute substantially to morbidity and mortality across all age groups. The frequency of these neoplasms is higher in children aged up to 5 years old, with most being malignant gliomas, germ-cell, and embryonal tumors. In adults, malignant CNS tumors, especially gliomas, are among the leading causes of death [31,34].

The basic criteria for the characterization of these entities have traditionally depended on histological, immunohistochemical, and cytological observations, often linked to their likeness to an alleged cell type of origin. The recognition of CNS tumors solely based on morphological features began to hinder the diagnosis of several subgroups and, hence, proper treatment. Eventually, as described in the 5th edition of the World Health Organization (WHO) CNS tumor classification (CNS5, 2021), this obstacle was overcome by incorporating molecular and genetic alterations into the diagnostic criteria. According to the current classification, six families of both benign and malignant tumors have emerged, comprising adult-type diffuse gliomas, pediatric-type diffuse low-grade gliomas (DLGG), pediatric-type diffuse high-grade gliomas (DHGG), circumscribed astrocytic gliomas, glioneuronal/neuronal tumors, and ependymal tumors [33,35].

The implementation of molecular assays in their diagnosis, such as DNA/RNA sequencing, genome-wide methylation profiling, quantitative PCR (qPCR), and FISH, has revealed a broad spectrum of genetic alterations in CNS tumors, encompassing point mutations, insertions and deletions, copy number changes, and gene rearrangements [36]. The genes that are more frequently affected are vital to cellular homeostasis. For instance, alterations in genes encoding for the phosphoinositide 3-kinase (PI3K), epidermal growth factor receptor (EGFR), V-Raf murine sarcoma viral oncogene homolog B (BRAF), platelet-derived growth factor receptor α (PDGFRA), and Met tyrosine-protein kinase (MET) lead to defective receptor tyrosine kinase signaling. The regulation of the cell cycle is also affected by mutations in the *p53*, *retinoblastoma susceptibility* (*RB1*), *cyclin-dependent kinase 4* (*CDK4*), *cyclin-dependent kinase inhibitor 2A* and *2B* (*CDKN2A* and *CDKN2B*), and *v-myb avian myeloblastosis viral oncogene homolog* (*MYB*) genes. Moreover, genetic changes in telomerase reverse transcriptase (TERT) and α-thalassemia intellectual disability X-linked (ATRX) genes affect the preservation of telomere integrity. Additionally, modifications in histone variants H3.1 and H3.3, predominantly the substitutions *K27M* and *G34V/R*, are implicated in abnormal chromatin arrangement and epigenetic regulation of gene expression [35,36,37,38]. Cell metabolism is also affected through the production of oncometabolites, such as 2-hydroxyglutarate (2-HG), which arises from mutations in the *isocitrate dehydrogenase* (*IDH*) gene [39]. The mutations in *IDH1* and *IDH2* often co-exist with concurrent deletion of chromosome arms 1p and 19q (1p/19q codeletion) and *TERT* alterations [40].

The treatment of CNS tumors continues to pose difficulties in both pediatric and adult age groups. More specifically, several parameters need to be considered in terms of tumor cell origin, location, genetic background, microenvironment and effective drug delivery. The standard clinical strategies, so far, involve surgery, radiation, and chemotherapy [30,41,42]. Over the last years, more targeted therapies, incorporating inhibitors, chimeric antigen receptor-T (CAR-T) cells, and vaccines, among others, have emerged. Regarding glioblastoma multiforme (GBM), the most frequent and aggressive form of glioma, immunotherapy may prove to be a promising treatment option [43]. Nonetheless, brain tumors still represent one of the main causes of cancer-related mortality, while survivors face a high risk of chronic health conditions, thus underscoring the pressing need for new treatment modalities [42,44].

### 2.1. MAPK Pathway Alterations in CNS Tumors

**Figure 2:**
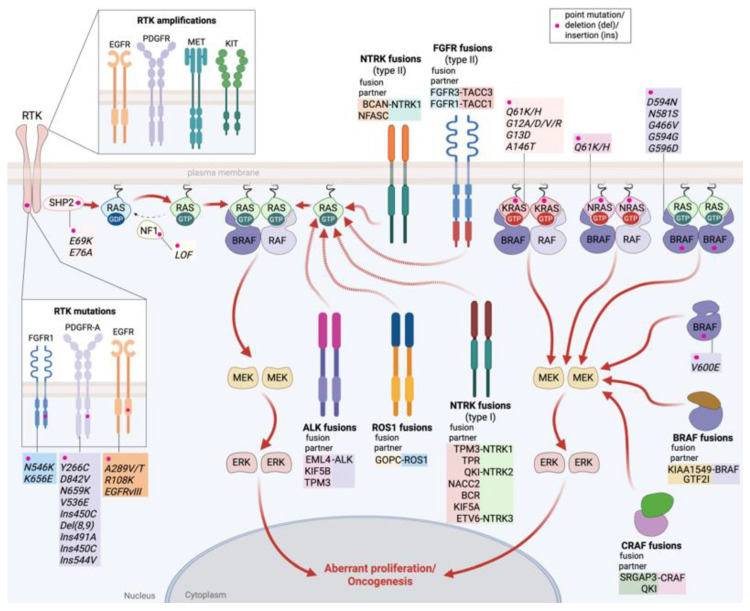
Representative alterations in the MAPK signaling cascade in central nervous system (CNS) tumors. Distinct genetic mechanisms drive aberrant signaling at multiple levels: (i) RTK amplifications, mutations, and fusions (e.g., *EGFR*, *PDGFRA*, *FGFR*, *MET*, *ALK*, *ROS1*, *NTRK*); (ii) activating *RAS* mutations in *KRAS* or *NRAS*, that impair GTP hydrolysis; (iii) oncogenic *RAF* alterations, most notably in *BRAF*, which include fusions such as *BRAF-KIAA1549* leading to constitutive activation, hotspot substitutions like *BRAF V600E* conferring to the oncoprotein strong monomeric kinase activity, and class III mutations (e.g., *BRAF G464E*, *D594G*) that produce kinase-impaired proteins dependent on upstream RAS or RTK signaling; and (iv) alterations in negative or positive regulators of MAPK signaling, such as *NF1 LOF* mutations and activating *SHP2* mutations. Collectively, these lesions converge on hyperactivation of the RAF–MEK–ERK axis, driving proliferation, survival, and therapeutic resistance in CNS tumors. Solid red arrows depict activation and dashed arrow depicts deactivation. EGFR, epidermal growth factor receptor; PDGFRA, platelet-derived growth factor receptor α; FGFR, fibroblast growth factor receptor; MET, mesenchymal–epithelial transition factor; ALK, anaplastic lymphoma kinase; ROS1, ROS proto-oncogene 1; NTRK, neurotrophic tyrosine receptor kinase; RAS, rat sarcoma viral oncogene; KRAS, Kirsten rat sarcoma viral oncogene homolog; NRAS, neuroblastoma rat sarcoma viral oncogene homolog, Harvey rat sarcoma viral oncogene homolog; RAF, rapidly accelerated fibrosarcoma; BRAF, V-Raf murine sarcoma viral oncogene homolog B; CRAF, proto-oncogene c-Raf; NF1, neurofibromin; SHP2, Src homology region 2 domain-containing phosphatase-2; MEK, mitogen-activated protein kinase; ERK, extracellular signal-regulated kinase; LOF loss-of-function; GTP, guanosine triphosphate; GDP, guanosine diphosphate. Created in BioRender. Adamopoulos, C. (2025) https://BioRender.com/krxcp45 (Assessed on 7 September 2025).

Aberrant activation of the MAPK pathway is a hallmark of several CNS tumors, frequently driven by genetic alterations such as point mutations, gene fusions, amplifications, or overexpression, most commonly involving RTKs, RAS, RAF, and regulators of the pathway such as SHP2 and NF1 [22,45,46] (Figure 2).

#### 2.1.1. Receptor Tyrosine Kinase (RTK) Alterations

RTKs are critical oncogenic drivers in gliomas, affected by diverse mechanisms, which include point mutations, gene amplifications, and chromosomal rearrangements that create fusion oncoproteins (Figure 2). These aberrations commonly confer ligand-independent kinase activation, leading to persistent MAPK pathway signaling [47]. For instance, EGFR alterations, most notably gene amplification and the *EGFRvIII* deletion variant, are prevalent in GBM and drive aggressive proliferation and therapeutic resistance [48]. Similarly, PDGFR-A amplification is characteristic of the proneural GBM subtype, while MET amplification and PDGFR overexpression also contribute to glioma pathogenesis [49,50] (Figure 2). Importantly, in pediatric gliomas, gene fusions involving *anaplastic lymphoma kinase* (*ALK*), *ROS proto-oncogene 1* (*ROS1*), *neurotrophic tyrosine receptor kinase* (*NTRK2*) and *MET* define a distinct, hemispheric high-grade subgroup with intermediate prognosis [51]. *Fibroblast growth factor receptor* (*FGFR*) gene family alterations, including *FGFR3–transforming acidic coiled-coil containing protein 3* (*TACC3*) fusions, constitute actionable drivers in a subset (3–5%) of GBM, producing fusion proteins that promote oncogenesis [52].

In pilocytic astrocytomas (PAs), fusions of the *NTRK2* gene, which encode for the tropomyosin receptor kinase B (TrkB) with either the transcriptional repressor nucleus Accumbens-associated protein 2 (NACC2), NACC2-NTRK2, or the pre-mRNA alternative splicing regulator Quaking homolog KH domain containing RNA binding (QKI), QKI-NTRK2, can induce MAPK pathway hyperactivity in a ligand-independent manner [53]. Additionally, in PA patients, the activating substitutions *N546K* and *K656E* in the *FGFR1* gene have been linked to elevated phosphorylated/activated ERK levels [54]. In a small number of infant-type hemispheric gliomas (IHGs), gene fusions involving *ALK*, *NTRK1*, and *ROS1* were detected without co-occurrence [54,55]. In the same study, a case of PA bore a fusion of *breakpoint cluster region* (*BCR*) and *NTRK2* genes (*BCR-NTRK2*), while a patient with pleomorphic xanthoastrocytoma (PXA) harbored the *tropomyosin 3* (*TPM3*)*-NTRK1* fusion. Moreover, two cases of gangliogliomas exhibited the fusion *FGFR3-TACC3* [54,55].

The localization of NTRK fusions varies depending on the fusion partner. Thus, when the 5′ fusion partner encodes a membrane or extracellular protein, such as the protein fusions of TrkA with the proteoglycan brevican (BCAN), BCAN–NTRK1, and the cell surface protein neurofascin (NFASC), NFASC–NTRK1, the fusion protein is membrane-associated (Type II) (Figure 2). Otherwise, when the fusion partners are cytosolic or nuclear, such as TPM3, QKI, Ets variant transcription factor 6 (ETV6), localization is cytoplasmic (Type I) (Figure 2). Yet all retain the NTRK kinase domain and drive constitutive MAPK signaling [56]. In GBM, Golgi-associated PDZ and coiled-coil motif-containing protein (GOPC)–ROS1 fusion proteins exhibit isoform-specific subcellular localization with the long form localizing to the Golgi, while the short form is cytoplasmic [57].

#### 2.1.2. RAS Alterations

All three RAS small GTPases, Kirsten rat sarcoma viral oncogene homolog (KRAS), neuroblastoma rat sarcoma viral oncogene homolog (NRAS) and Harvey rat sarcoma viral oncogene homolog (HRAS), function as molecular switches that alternate from their inactive GDP-bound state to their active GTP-bound state (Figure 1). Oncogenic *RAS* mutations, typically missense substitutions at hotspot codons 12, 13, or 61, largely impair intrinsic GTP hydrolysis and lock RAS in its active form, resulting in constitutive downstream signaling [58] (Figure 2). While *RAS* mutations are among the most common oncogenic drivers in many cancers, they are relatively rare in gliomas. Nevertheless, accumulating evidence indicates that RAS alterations can contribute to gliomagenesis across different subtypes, either as point mutations or gene copy number gains, often cooperating with other oncogenic events to sustain tumor growth and progression [59].

In two distinct cases of PXA, a *KRAS* mutation at codon 61 was detected, which codes for glutamine in position 61 of KRAS protein [60]. More specifically, in one case, glutamine was substituted by lysine (*Q61K*), while in the other, it was replaced by histidine (*Q61H*). Interestingly, these two cases were the first in which a *KRAS* mutation was detected in PXA patients [60]. Despite its rarity, a case of ganglioma with an *NRAS* mutation was reported among a heterogeneous group of 30 patients with infantile CNS tumors [54,55]. Oncogenic missense mutations in *KRAS* and *NRAS* were present in 8 patients with *IDH*-mutant astrocytomas, including *G12A/D/V*, *G13D*, *D33E*, *A146T*, and *K117N* substitutions. In the same cohort, 3 tumor samples reported a high increase in *KRAS* gene copies [25,54,55].

#### 2.1.3. MAPK Pathway Regulators Alterations

Beyond RTKs and RAS itself, several intracellular modulators of MAPK signaling are altered in CNS tumors. The most relevant include neurofibromin (NF1) and the associated sprouty-related EVH1 domain-containing protein 1 (SPRED1) and leucine zipper-like transcription regulator 1 (LZTR1), and Src homology region 2 domain-containing phosphatase-2 (SHP2). NF1 acts as a tumor suppressor by accelerating RAS GTP hydrolysis, a process facilitated by SPRED1, which recruits NF1 to the plasma membrane [24,61]. In PXAs, *NF1* mutations were reported in 3 of 13 cases, including two missense and one truncating variant [58]. In *IDH*-mutant astrocytomas, *NF1* alterations occurred in 17 of 27 cases, while *LZTR1* mutations (nonsense, frameshift, splice site, or missense) were also observed, consistent with loss of its role in targeting RAS proteins, among others, for ubiquitin-mediated degradation [62]. Inactivating SPRED1 lesions, including biallelic deletion and frameshift changes, have also been described in this tumor type [24].

On the other hand, SHP2 is a positive effector that promotes MAPK pathway activation, as it functions as an adaptor protein and phosphatase downstream of multiple RTKs (Figure 1). Gain-of-function mutations, particularly *E69K* and *E76A*, enhance its activity, facilitating sustained RAS/MAPK signaling in PA [54,63] (Figure 2).

#### 2.1.4. RAF Alterations

Within the RAF family of serine/threonine kinases, BRAF is the predominant oncogenic driver in cancers, including gliomas, followed less frequently by CRAF and only rarely by ARAF. RAF proteins under physiological conditions signal as BRAF homodimers or BRAF-CRAF heterodimers, constituting the most common and biologically relevant signaling forms [64,65] (Figure 1). Oncogenic BRAF alterations, through gene fusions or hotspot mutations, result in sustained constitutive downstream MAPK activation.

The most prevalent mutation in pilocytic astrocytomas is a genomic rearrangement that leads to the fusion of the *KIAA1549* and *BRAF* genes. The structural rearrangement involves the duplication of a DNA segment between the *KIAA1549* 5′-end and *BRAF* 3′-end genes in the 7q34 chromosomal region, spanning approximately 2 Mb. From this event, five different in-frame variants have been identified: *KIAA1549^ex16^-BRAF^ex9^*, *KIAA1549^ex15^-BRAF^ex9^*, *KIAA1549^ex19^-BRAF^ex9^*, *KIAA1549^ex16^-BRAF^ex11^*, and *KIAA1549^ex18^-BRAF^ex10^*. All the resulting chimeric proteins exhibit constitutive activation, as they all lack the N-terminal domain responsible for the autoregulation of BRAF, due to substitution from KIAA1549. At the same time, they maintain the kinase domain of BRAF [66,67,68,69]. Interestingly, in a small cohort of PAs several other fusion partners have been identified for BRAF, including family with sequence similarity 131 member B (FAM131B), ring finger protein 130 (RNF130), chloride voltage-gated channel 6 (CLCN6), makorin ring finger protein 1 (MKRN1), guanine nucleotide-binding protein subunit alpha-11 (GNA11), quaking homolog KH domain RNA binding protein (QKI), fizzy and cell division cycle 20 related 1 (FZR1), microtubule actin crosslinking factor 1 (MACF1), and general transcription factor II-I (GTF2I). Although biologically and functionally disparate, these fusion partners render domains that converge on the same outcome: hyperactivation of BRAF and its downstream signaling [24,54,70,71].

Regarding *BRAF* mutations, the second most common alteration detected in PAs is a substitution of valine in position 600 by glutamic acid, which results in *BRAF V600E* the most frequent *BRAF* mutation in human cancers [69,72,73]. This point mutation disrupts the regulatory conformation of BRAF, resulting in loss of its N-terminal autoinhibitory domain and conferring monomeric kinase activity with hyperactivation of the MAPK pathway (Figure 2) [72,73]. *BRAF V600E* is observed across several glioma subtypes. In a study by Zou et al., who evaluated mutations in a cohort of 13 PXA patients using next-generation sequencing, the *BRAF V600E* mutation was present in 38% of the cases [60]. In a comprehensive analysis of 30 infantile (<12 months old) CNS tumors, 7/10 cases of desmoplastic infantile ganglioglioma (DIG) harbored alterations in BRAF (5 mutations, 1 duplication and 1 fusion), 1/2 cases of PXA carried the *CAP-Gly domain containing linker protein 2* (*CLIP2*)-*BRAF* fusion, 1/2 cases of PA had the *KIAA1549-BRAF* fusion, and a single case of DLGG was *BRAF V600E*-mutant [55]. Tumors from 3 young-adult patients with *IDG*-mutant astrocytomas possessed a rare in-frame *protein tyrosine phosphatase receptor type Z1* (*PTPRZ1*)-*BRAF* gene fusion and two class III *BRAF* mutations, the substitutions *G464E* and *D594G* [25]. *G464E* affects the kinase domain of BRAF, producing a kinase-impaired protein that requires RAS activation, whereas *D594G* affects the activation segment of BRAF, resulting in a kinase-dead protein, both relying on upstream RAS/RTK activity [74,75].

In the context of chimeric proteins, the gene encoding CRAF protein (*CRAF* or *RAF1*) has been observed to fuse either with the *nuclear transcription factor 1A* (*NF1A*) or *SLIT-ROBO Rho GTPase activating protein 3* (*SRGAP3*) gene in some rare PA case reports at chromosomal regions 1q31.3 and 3p25, respectively. The end-product of both translocations is an oncoprotein that augments constitutive MAPK pathway activation [68,76].

Alterations are not limited to BRAF. In the study of Tauziède-Espariat et al., 2/30 infantile patients with tumors characterized as DIGs carried *CRAF* fusions, particularly one of these cases presented with the protein kinase cAMP-dependent type II regulatory subunit a (*PRKAR2A*)-*RAF1* fusion [55]. *CRAF* fusions have been described in rare PAs, involving the *nuclear transcription factor 1A* (*NF1A*) or *SLIT-ROBO Rho GTPase activating protein 3* (*SRGAP3*) as fusion partners [68,76]. In infantile DIGs, *CRAF* fusions have also been identified, including a protein kinase cAMP-dependent type II regulatory subunit α (PRKAR2A)-CRAF chimera [55]. Additional *RAF1* fusions, contributing to constitutive MAPK activity, have been documented across gliomas and other tumor types [77].

## 3. RAS/MAPK Pathway Inhibitors in CNS Tumors

The high frequency of activating mutations and other genetic alterations in the RAS/RAF/MEK/ERK signaling axis and its subsequent hyperactivation has highlighted their association with cancer development and progression [78]. Consequently, components of this pathway have become promising therapeutic targets through their selective inactivation by small-molecule inhibitors. In addition, alternative medicinal chemistry strategies with the development of PROTACs or molecular glues have emerged. All these targeting efforts have been directed towards CNS tumors as well [79].

### 3.1. RAF Inhibitors

The high frequency of the *BRAF V600E* mutation, accounting for 95% of all BRAF mutations, and the increased kinase activity of the BRAF V600E oncoprotein made it an ideal pharmacological target for small-molecule inhibitors. This led to the development of the first- and eventually the more selective second-generation RAF inhibitors targeting the mutant-BRAF kinase [72,73]. The increased selectivity for the monomeric mutated BRAF versus the dimeric wild-type BRAF is the basis of the high therapeutic index of the second-generation RAF inhibitors [74]. These discoveries resulted in the FDA’s approval of vemurafenib in 2011 and dabrafenib in 2013, as single agents, for the treatment of metastatic *BRAF V600E*-mutant melanoma [72,73]. Since then, combination therapies using the RAF inhibitors vemurafenib, dabrafenib and encorafenib, along with the MEK inhibitors trametinib, cobimetinib and binimetinib or the EGFR inhibitor cetuximab, have gained FDA approvals in subsequent years for other types of cancer, harboring the *BRAF V600E* mutation [72,73,80]. However, the effectiveness of these monomer-selective RAF inhibitors is often hindered by the development of adaptive resistance, primarily mediated by the formation of RAF dimers, through MAPK-pathway reactivation because of the relief of negative feedback or via secondary genetic alterations [72,73,74]. To overcome the dimer-forming resistance mechanisms, next-generation RAF inhibitors that target the dimeric form of RAF have been developed [72,73,74]. Recently, one such inhibitor, tovorafenib, was clinically approved for treating pediatric patients with low-grade glioma (LGG) carrying genetic alterations in the *BRAF* gene [26].

#### 3.1.1. Vemurafenib

Vemurafenib is a selective BRAF V600E inhibitor that competes with ATP binding, thus preventing downstream MEK activation. It exhibits limited penetration across the BBB, which restricts its efficacy in primary brain tumors (Table 1) [81]. Initially approved for metastatic melanoma, vemurafenib has shown partial efficacy in *BRAF*-mutant gliomas in case series and small trials [82,83,84]. Responses tend to be short-lived due to the development of adaptive resistance and insufficient CNS concentrations. Common adverse effects include rash, joint pain, fatigue and paradoxical activation of wild-type BRAF leading to secondary malignancies like squamous cell carcinoma [82,83,84].

#### 3.1.2. Dabrafenib

Dabrafenib is another selective BRAF V600E inhibitor with superior BBB penetration and a more favorable safety profile compared to vemurafenib [85]. Clinical trials have demonstrated that dabrafenib is effective in pediatric patients with *BRAF*-mutant LGGs, leading to tumor regression and improved progression-free survival [86]. On 16 March 2023, dabrafenib combined with the MEK inhibitor trametinib gained FDA approval for pediatric *BRAF V600E*-mutant LGGs (Table 1) [86]. This synergistic regimen shows improved tolerability, with fewer secondary skin malignancies when used in combination therapy. Patients may exhibit pyrexia, fatigue, skin rash and arthralgia [87].

#### 3.1.3. Encorafenib

Encorafenib is a newer first-generation BRAF V600E inhibitor developed to reduce paradoxical activation and enhance the duration of response [88]. While it is primarily used in melanoma and colorectal cancer, preclinical studies are investigating its use in brain tumors [89]. Although encorafenib features a longer dissociation half-life from BRAF V600E and potentially better pharmacodynamic suppression of MAPK signaling, its efficacy in CNS tumors may be limited by its lower BBB penetration (Table 1) [90].

#### 3.1.4. NST-628

NST-628 is a non-degrading molecular glue that binds to both RAF and MEK proteins, stabilizing their complex in a way that prevents MEK phosphorylation by RAF, effectively blocking downstream signaling [91]. This mode of action avoids resistance mechanisms common in traditional kinase inhibitors. NST-628 inhibits all RAF isoforms (ARAF, BRAF, CRAF) and works across multiple *RAS*- and *RAF*-mutant cancers, including those resistant to existing therapies. Unlike many inhibitors, NST-628 is brain-penetrant, making it potentially effective against CNS tumors (Table 1). The compound induces long-lasting suppression of the MAPK pathway in both in vitro and, also, in vivo models, including mouse xenografts and organoids derived from human tumors. As a result, due to its broad activity, resistance-evasion capacity, and brain penetration, NST-628 shows promise for treating a wide range of *RAS*- and *RAF*-driven CNS cancers, including those with KRAS, NRAS, or BRAF mutations [91].

### 3.2. MEK Inhibitors

Selective MEK inhibitors have been developed to effectively block the MAPK pathway activation, especially after its reactivation due to the relieved negative feedback mechanisms following BRAF inhibitor therapy [72,73,74]. Thus, combinatorial targeting of MEK inhibitors (trametinib, cobimetinib, binimetinib) with RAF inhibitors (vemurafenib, dabrafenib, encorafenib) has been FDA-approved, from 2014 to 2018, for patients with metastatic melanoma, non-small cell lung cancer (NSCLC), and anaplastic thyroid cancer carrying the *BRAF V600E* mutation [72,73,92,93,94]. In 2021, the FDA approved the MEK inhibitor selumetinib for pediatric patients with neurofibromatosis type 1, a genetic disorder in which *NF1* loss predisposes to peripheral nerve sheath tumors and other cancers (Table 1) [95,96]. Most MEK inhibitors disrupt the formation of the RAF-MEK complex, inhibiting MEK phosphorylation and activation [97].

#### 3.2.1. Selumetinib

Selumetinib is an allosteric MEK inhibitor that prevents ERK activation and has demonstrated significant efficacy in *NF1*-associated pLGGs [98], as well as in non-*NF1*-associated pLGGs [99], including disease stabilization and, in some cases, prolonged disease control (Table 1). Furthermore, it is an orphan drug designation for *NF1*-altered gliomas [100]. Generally, it is well tolerated in children, but adverse effects, like gastrointestinal symptoms, skin rash, rare cardiomyopathy and ocular toxicity may arise [98,99,100]. Ongoing clinical trials comparing selumetinib with conventional chemotherapy in both *NF1*-associated and non-*NF1* pLGG will further clarify its therapeutic value and long-term safety. Notably, emerging evidence indicates that a subset of patients can maintain durable progression-free survival (PFS) even after treatment cessation, underscoring the potential of MEK inhibition as a promising disease control strategy [99,100].

#### 3.2.2. Trametinib

Trametinib is a potent selective allosteric MEK inhibitor, often used in combination with dabrafenib [92,93,94,97,101]. Approved in combination for *BRAF V600E*-mutant tumors, trametinib enhances efficacy and reduces adverse effects such as secondary skin cancers [92,93,94,101,102]. Current results from an ongoing clinical trial demonstrate significant clinical benefit to the majority of both pLGG and plexiform neurofibromas (PNs) patients, including measurable responses and prolonged PFS (Table 1) [102]. Common adverse effects are diarrhea, skin rash, fatigue, and hypertension [102].

#### 3.2.3. Binimetinib and Cobimetinib

The MEK inhibitors binimetinib and cobimetinib have been tested primarily in non-CNS malignancies but are currently under investigation in gliomas [89]. Ongoing trials are evaluating their BBB permeability and potential in combination with BRAF and mTOR inhibitors, supported by favorable pharmacokinetics and CNS bioavailability [103]. Binimetinib is currently under clinical investigation in brain tumors, including high-grade glioma (HGG) [89]. Cobimetinib provided efficacy when tested in combination with vemurafenib in a refractory case of *BRAF V600E*-mutated ganglioglioma [104]. Its role in neuro-oncology, however, remains to be fully defined. Given their pharmacologic profiles, both agents represent promising candidates for rational combination strategies targeting multiple signaling pathways in gliomas.

#### 3.2.4. Mirdametinib

Mirdametinib is an orally bioavailable MEK inhibitor that has recently achieved its first regulatory approval in the United States for the treatment of symptomatic, inoperable *NF1*-associated PNs in both adult and pediatric patients (Table 1) [105]. Beyond neurofibromatosis type 1, ongoing trials are evaluating its efficacy in pLGGs and other RAS/MAPK-driven tumors, providing a rationale for its potential application in primary brain tumors [105,106]. With established clinical activity in *NF1*-associated CNS tumors and a favorable oral dosing profile, mirdametinib represents a promising candidate for expanding MEK-directed strategies in neuro-oncology [105,106].

### 3.3. ERK Inhibitors

Although mutations in ERK proteins are rare, selective ERK inhibitors are under preclinical development, given that ERK is the terminal kinase of the RAS/RAF/MEK/ERK signaling cascade, seeking a more durable inhibitory response [107]. These agents are particularly promising in tumors that develop resistance to BRAF and/or MEK inhibitors.

#### Ulixertinib (BVD-523)

Ulixertinib is an oral, ATP-competitive ERK inhibitor that has demonstrated preclinical efficacy in various tumor models, including those resistant to BRAF and MEK inhibitors. Phase I clinical trials have shown acceptable tolerability and preliminary antitumor activity in patients with advanced solid tumors harboring MAPK pathway alterations. In gliomas, its ability to cross the BBB and suppress ERK-driven transcription makes it a promising candidate, although its application is under exploration (Table 1) [108]. Elevated liver enzymes, diarrhea and fatigue are the main observed side effects of this drug [108].

### 3.4. SHP2 Protein Inhibitors

Alongside direct targeting of the RAS/RAF/MEK/ERK axis components, selective inhibitors have been developed against the SHP2 phosphatase (e.g., TNO155 and RMC-4630), which block upstream activation of RAS by inhibiting the GRB2-SOS1 interaction [109] (Figure 1). Specifically, a study has shown that SHP2 inhibition, using the SHP2 inhibitor SHP099, could efficiently reduce RAS-GTP loading, block RAS-mediated RAF/MEK/ERK signaling and abrogate tumor growth in *NF1*-malignant peripheral nerve sheath tumors (MPNSTs) (Table 1) [110]. Furthermore, combining SHP2 inhibition treatment with hydroxychloroquine (HQ), a pharmacological inhibitor of autophagy, showed enhanced effectiveness in mouse and human *NF1*-MPNST models [110]. Additionally, Sang and colleagues examined the efficacy of SHP099 in GBM with activated PDGFR-A. SHP099 exhibited antitumor activity either as a single agent or in combination with temozolomide (TMZ) and provided significant survival benefits for GBM tumor xenograft-bearing animals [111].

### 3.5. Combinatorial Therapies

Combined inhibition of multiple MAPK pathway components enhances treatment efficacy and reduces the risk of resistance or overcomes the already developed adaptive resistance [72,74,97,112,113]. BRAF plus MEK inhibitor is the most validated combination, especially in GBM and in pediatric LGG [114,115]. It delays resistance, lowers toxicity, and provides better PFS compared to monotherapy. Furthermore, ongoing trials are exploring a multi-combinatorial strategy of BRAF, MEK, and AKT inhibitors [116,117]. Lastly, MAPK inhibitors could combine with immunotherapy, given that MAPK inhibition may increase immune recognition, making combination with immune checkpoint inhibitors (ICIs) a promising therapeutic avenue [118,119].

### 3.6. Clinical Application and Efficacy

#### 3.6.1. Pediatric Low-Grade Glioma (LGG)

Pediatric LGGs are the most frequent pediatric brain tumors and are characterized by indolent growth but can cause significant morbidity. Molecular profiling has revealed that most of these tumors harbor MAPK pathway alterations. The combination of dabrafenib and trametinib has demonstrated remarkable efficacy in pediatric LGGs with *BRAF V600E* mutations [101,114,115]. In clinical trials, response rates exceeded 70%, and the combination was associated with PFS and tolerable side effects [114,115]. Selumetinib has also shown clinical benefit in *NF1*-associated pediatric LGGs [98]. Results from Phase II trials indicated that selumetinib led to tumor shrinkage and visual improvement in children with optic pathway gliomas [99,100].

#### 3.6.2. High-Grade Glioma (HGG)

In HGG, the MAPK pathway is often only one of many dysregulated networks, and monotherapy with BRAF or MEK inhibitors has generally been less effective [120]. However, in select cases, such as *BRAF V600E*-positive GBM, targeted therapies have resulted in durable responses [101,121]. Combination therapy is currently under active investigation in clinical trials, including regimens that pair MAPK inhibitors with other targeted or immunotherapeutic agents [122,123]. Specifically, Arbour and colleagues reported an 18-year-old female with a grade III PXA treated upfront with dabrafenib and trametinib [122]. Also, Fusco et al. describe a similar case of a 19-year-old male patient with grade III PXA, who achieved durable PFS with BRAF and MEK inhibitors combination [123].

#### 3.6.3. Ganglioglioma

Gangliogliomas are usually low-grade brain tumors containing both neuronal and glial elements, most often occurring in children and young adults. A large proportion of these tumors harbor activating mutations in the MAPK signaling pathway, particularly BRAF V600E, which renders them responsive to MEK inhibitors [124]. Nonetheless, some gangliogliomas lack identifiable MAPK pathway alterations and therefore have not traditionally been considered candidates for MEK-targeted therapy. Interestingly, a recent report described a young adult patient with ganglioglioma who did not carry MAPK pathway mutations but achieved a marked and durable response to the MEK inhibitor trametinib [125].

#### 3.6.4. Medulloblastoma

Medulloblastoma is a common malignant pediatric brain tumor. While existing treatments can be effective, they often cause significant long-term side effects [126]. A major clinical challenge is resistance to therapy and recurrence, often driven by tumor stem-like cells [127]. The protein BMI1, a known regulator of stem cell renewal and tumorigenesis, is overexpressed in medulloblastoma and supports tumor growth [128]. A study investigates whether targeting BMI1, alone or in combination with MAPK/ERK pathway inhibitors, could be an effective treatment strategy against medulloblastoma [129]. The study used the PD325901, a MEK inhibitor that blocks ERK phosphorylation, as the MAPK/ERK pathway inhibitor in combination with BMI1 inhibition to evaluate synergistic effects on medulloblastoma cells [129].

### 3.7. Current and Ongoing Clinical Trials

Several ongoing clinical trials assess the MAPK inhibition in different CNS tumor types (Table 2). Selectively, some of them include the evaluation of the dabrafenib plus trametinib combination in pLGGs [114], which paved the way for the FDA approval of this combination for treatment, the investigation of the effectiveness of selumetinib in *NF1*-associated gliomas [96], and the study of the role of tovorafenib in relapsed pLGG with BRAF alterations (FIREFLY-1/NCT04775485) [130]. These studies are refining indications, dosing and combinations, and will help define future standard-of-care approaches.

## 4. Therapeutic Challenges of Targeting the MAPK Pathway in Brain Tumors

### 4.1. Blood–Brain Barrier (BBB) and Drug Delivery Limitations

The therapeutic management of intracranial tumors such as gliomas, meningiomas, pituitary adenomas and craniopharyngiomas is limited by the presence of both the BBB and the blood–tumor barrier (BTB). BBB’s fundamental role through its high selectivity is to maintain cerebral homeostasis, but at the same time, it restricts the entry of many pharmacological agents, especially large or hydrophilic molecules [131,132,133]. In contrast, the BTB, which arises from abnormal tumor-induced angiogenesis, displays heterogeneous permeability [134]. This results in uneven intratumoral drug distribution, particularly in aggressive tumors such as GBM and craniopharyngiomas. These anatomical and physiological characteristics affect the uniform delivery and eventually the efficacy of systemically administered drugs [28,135]. Thus, effective brain tumor treatment requires the development of compounds that both target oncogenic signaling pathways and, at the same time, achieve adequate penetration through the BBB. However, even small, lipophilic molecules can fail to accumulate sufficiently in the CNS due to active efflux mechanisms mediated by ATP-binding cassette (ABC) transporters, which include the P-glycoprotein (P-gp/ABCB1) and breast cancer resistance protein (BCRP/ABCG2) [136,137]. Such transporters are located at the BBB/BTB interface and within tumor cells and contribute to chemoresistance by actively extruding therapeutic agents from the brain parenchyma [138]. Importantly, ABC transporter expression is heterogeneous across tumor subtypes and can be upregulated in response to treatment. For instance, exposure to doxorubicin has been demonstrated to induce the expression of multiple transporters, such as P-gp, BCRP, MRP-1, -2, -3, and -6, in gliomas, further compounding resistance [139].

Many RTK inhibitors, including erlotinib, gefitinib, and afatinib, are known substrates of P-gp and BCRP, restricting their CNS bioavailability [140,141,142]. Certain compounds, such as sunitinib and sorafenib [143], and third-generation EGFR inhibitors, such as osimertinib, rociletinib, and HM61713, demonstrate improved BBB penetration and activity against resistance mutations like *EGFR T790M* challenges persist [142]. Similar pharmacokinetic barriers are encountered with RAF inhibitors, like vemurafenib and dabrafenib, and MEK inhibitors like trametinib, cobimetinib, binimetinib, selumetinib and pimasertib, many of which are subject to efflux via P-gp and BCRP. Other MEK inhibitors such as PD0325901 and E6201 have shown favorable BBB permeability in preclinical studies [144,145]. Furthermore, newer-generation RAF inhibitors, including dabrafenib, encorafenib, and the molecular glue, dual RAF-MEK inhibitor NST-628, have demonstrated enhanced CNS distribution, thus improving their therapeutic usage in intracranial malignancies [87,89,91]. Compounds that target KRAS G12C, like sotorasib and adagrasib, and ERK, like ulixertinib, are in active clinical evaluation. However, their pharmacokinetic properties and association with efflux transporters are not yet defined [108,146,147]. Several innovative drug delivery strategies are currently being explored, including nanoparticles, focused ultrasound-mediated BBB disruption and convection-enhanced delivery [28].

### 4.2. Tumor Heterogeneity and Resistance Mechanisms in MAPK Pathway-Targeted Therapies

Genetic alterations in the MAPK signaling pathway vary across different brain tumor types, affecting disease progression and therapeutic response. Hyperactivating mutations such as *BRAF V600E* and *BRAF–KIAA1549* fusions are frequently observed in pediatric and adult low-grade gliomas, such as PAs, gangliogliomas, and PXAs [54,148]. Mutations in genes including *ROS1*, *ALK*, *NF1*, *KRAS*, *MEK*, and *CRAF* have been identified across glioma subtypes, highlighting the necessity for molecular classification beyond traditional histopathology [35,149,150]. Additionally, fusions involving *FGFR* and *NTRK* family genes and fusions and amplifications in *ALK*, *ROS1*, and *MET*, have been detected predominantly in pediatric brain tumors [51,151,152]. Mutations in *PIK3CA* and *AKT1* are frequent in meningiomas, whereas activating mutations affecting the RAS/RAF/MEK/ERK, PI3K/AKT, and Wnt pathways have been described in pituitary adenomas [153,154]. Apart from primary CNS tumors, genetic alterations within the MAPK pathway, such as *RTK* and *BRAF* mutations, are often observed in brain metastases originating from lung, breast, and melanoma primary cancers [155]. Despite the considerable therapeutic advances of the MAPK pathway-directed targeted therapies, their clinical success is frequently limited by the development of drug resistance [22,156]. These resistance mechanisms often include compensatory activation of parallel signaling cascades like PI3K/AKT/mTOR pathway [72,157,158]. Furthermore, reactivation and/or hyperactivation of the MAPK pathway through the relief of negative feedback loops upon treatment with MAPK inhibitors, fosters epigenetic reprogramming by inducing expression of key transcription factors associated with cellular stemness and mesenchymal transition. This process involves chromatin remodeling, enhancer reconfiguration, and rewiring of the transcription factor network, including the transcription factors SOX2, OLIG2, STAT3, KLF4, and NOTCH, promoting therapeutic evasion and adaptive resistance [72,73,74,157,158,159,160].

### 4.3. Tumor Microenvironment (TME)

The TME functions as a dynamic ecological system that actively shapes tumor evolution, therapeutic response, and resistance through complex and reciprocal interactions between tumor cells and their surrounding stromal and immune compartments [161]. Genetic and epigenetic alterations influence the transcriptional and secretory programs of cancer cells, thereby reprogramming the TME, which in turn contributes to the emergence of resistance to MAPK pathway inhibitors. Certain oncogenic mutations in both the RAS/MAPK and PI3K/AKT signaling pathways can support an immunosuppressive TME [162,163]. Moreover, in GBM, elevated levels of phosphorylated ERK have been associated with an increased TME infiltration by M2-type tumor-associated macrophages (TAMs) [164]. This altered microenvironment corresponds to “cold tumors,” characterized by minimal infiltration of immune cells and poor response to immunotherapies [165]. Moreover, sustained MAPK pathway activation can induce a senescence-associated secretory phenotype (SASP) that further modifies the TME, promoting the secretion of cytokines, chemokines and growth factors that enhance cancer cell viability and foster therapeutic resistance [166]. In *BRAF V600E*-mutant HGG, dual BRAF and MEK inhibition affects glioma plasticity, promoting an immunomodulatory phenotype with elevated PD-L1 expression and improving the synergy with ICIs [167]. Furthermore, TME can drive resistance upon combinatorial BRAF inhibitor and ICI treatment in brain metastatic acral melanoma [168]. A comprehensive understanding of the effects of the MAPK pathway inhibition in TME, along with tumor cells, will allow the rational design of novel therapeutic strategies suitable for brain tumors [29].

### 4.4. Toxicities Associated with MAPK Pathway Inhibitors

Targeted inhibition of the MAPK signaling cascade has significantly improved clinical outcomes in subsets of brain tumor patients. However, it is often associated with adverse effects and toxicities that affect treatment tolerability and limit long-term utilization [169,170,171,172,173,174,175,176,177]. BRAF inhibitors such as dabrafenib and vemurafenib are most commonly associated with cutaneous toxicities, including follicular or acneiform eruptions, xerosis, fatigue, and photosensitivity. While most dermatologic side effects are mild and manageable, treatment discontinuation may be required in cases of severe toxicity [169,170,171,172]. MEK inhibitors, including trametinib and selumetinib, also frequently cause dermatologic adverse events (rash, xerosis, paronychia). In addition, systemic toxicities such as fatigue and cardiovascular complications, including hypertension and bradycardia, have been reported. Given the risk of cardiotoxicity, especially in pediatric patients with CNS tumors, routine cardiac monitoring is strongly recommended [173,174]. Trk inhibitors, such as larotrectinib and entrectinib, target aberrant activation of Trk receptors resulting from *NTRK* gene fusions. Although generally well tolerated, these compounds have been linked to a spectrum of adverse effects, including gastrointestinal symptoms such as nausea, vomiting, diarrhea, hepatotoxicity, peripheral edema, cutaneous rashes, cardiac dysfunction, and neurological effects including dizziness, headache, and peripheral neuropathy [152,175,176,177].

## 5. Conclusions

The therapeutic targeting of the MAPK signaling pathway has clinical benefits in various CNS tumors, particularly in pLGGs that express the *BRAF V600E* mutation [113]. Monotherapy with BRAF or MEK inhibitors is often associated with drug resistance and substantial toxicities, while combination strategies, especially dual inhibition of RAF and MEK, have demonstrated superior efficacy [97,170]. For instance, the dabrafenib–trametinib combination has received FDA approval for *BRAF V600E*-mutant low-grade gliomas [154,178]. Drug resistance is frequently caused by reactivation of the RAF/MEK/ERK axis and alternative escape mechanisms from MAPK inhibition such as enhanced PI3K/AKT/mTOR signaling [79]. As a result, current efforts in preclinical models and early-phase clinical trials (e.g., NCT02023905, NCT02133183) focus on multi-targeted strategies, combining MAPK pathway inhibitors with inhibitors targeting parallel signaling pathways such as PI3K/AKT/mTOR. Resistance to MAPK inhibition may also arise through mechanisms that bypass the main signaling pathway, including activating mutations in PI3KC, AKT-mediated feedback loops, *PTEN* loss, mTOR upregulation, and autophagy-associated survival responses [22]. Therapeutic approaches focus on modulating apoptosis, disrupting tumor-associated metabolic reprogramming, regulating autophagy, and inhibiting phenotypic plasticity to enhance treatment efficacy [22,179,180,181]. Novel strategies aiming to reverse the immunosuppressive TME [182]. In this context, current approaches include the assessment of CAR T cells targeting tumor-associated antigens such as IL-13Rα2 and EGFRvIII, as well as inhibitors of PD-1/PD-L1 axis [183,184]. Consistently, PD-L1 overexpression in GBM has been associated with poor clinical outcomes [184,185,186]. Blockade of PD-1/PD-L1 interaction aims to suppress the PD-1-mediated inhibitory signaling, restore cytotoxic T-cell function and enhance anti-tumor immunity [187]. Current clinical trials assessing combinatorial immunotherapies involving ICIs, CAR T cells, and anti-angiogenic drugs (e.g., bevacizumab), showing promising preliminary results [28,188,189]. Combination approaches involving immunotherapies with MAPK pathway inhibition are being evaluated mostly to overcome resistance mechanisms [189,190]. Cytokine therapies are being explored for their potential to reactivate immune function within the TME. Such therapies have shown promise in augmenting immune responses without significant toxicities [188,191,192]. Moreover, inhibitors focused on metabolic reprogramming at the *IDH1/2*-mutant gliomas are under investigation [193]. Possible synergies of all these strategies with MAPK pathway inhibition in certain contexts could be proven highly beneficial, providing both sustained tumor suppression and enhanced TME immunomodulation.

Despite meaningful progress, the clinical management of high-grade brain tumors, such as GBM, continues to be hindered by tumor heterogeneity, adaptive resistance mechanisms, and the restrictive nature of the BBB. Moving forward, multimodal therapeutic strategies that address both the tumor and its surrounding microenvironment, along with personalized molecular profiling, will be critical for improving survival outcomes. The future of MAPK-targeted therapy in CNS tumors lies in precision medicine, with treatment paradigms tailored to each patient’s unique molecular and immunological landscape.

## Figures and Tables

**Table 1:** MAPK pathway inhibitors in central nervous system (CNS) tumors: molecular targets, tumor indications, and representative clinical outcomes. LGG, low-grade glioma; HGG, high-grade glioma; PXA, pleomorphic xanthoastrocytoma; GBM, glioblastoma multiforme; NF1, neurofibromin; OP, optic pathway; NF1-MPNST, NF1-associated malignant peripheral nerve sheath tumor; PN, plexiform neurofibroma; ORR, overall response rate; PFS, progression-free survival; FDA, Food and Drug Administration; BBB, blood–brain barrier; CNS, central nervous system; TMZ, temozolomide.

Drug/Strategy	Molecular Target(s)	CNS Tumor Type(s)	Key Clinical Findings	Selected Trials
Dabrafenib + Trametinib	BRAF V600E + MEK1/2	Pediatric LGG, HGG, PXA, GBM	ORR > 70% in BRAF V600E pLGG; significant PFS improvement; FDA-approved for pLGG (2023)	NCT07110246, NCT03919071
Vemurafenib	BRAF V600E	Glioma, PXA	Partial responses; limited durability due to resistance; modest BBB penetration	NCT01748149
Encorafenib (+ MEK inhibitors)	BRAF V600E	Glioma (investigational)	Improved pharmacodynamics vs. vemurafenib; CNS efficacy under study	NCT03973918
Selumetinib	MEK1/2	NF1-associated pLGG, OP glioma	Tumor shrinkage and visual improvement; durable disease control; FDA-approved for NF1 tumors	NCT01089101, NCT03871257
Trametinib	MEK1/2	pLGG, NF1 tumors, PXA	Clinical benefit in pLGG and PNs; enhanced efficacy with dabrafenib	NCT03363217
Mirdametinib	MEK1/2	NF1 tumors, pLGG	Recently FDA-approved for NF1-associated PN; promising CNS activity	NCT04923126
Tovorafenib	RAF	Relapsed pLGG with BRAF alterations	High response rate; effective in BRAF-fusion tumors; FDA-approved 2024	FIREFLY-1/NCT04775485
NST-628	RAF-MEK molecular glue	RAS/RAF-mutant gliomas	Potent, brain-penetrant MAPK suppression; preclinical efficacy	Preclinical
Ulixertinib	ERK1/2	Advanced glioma (investigational)	Activity in BRAF/MEK-resistant tumors; BBB penetration	NCT01781429
SHP2 inhibitors (TNO155, RMC-4630)	SHP2	GBM, NF1-MPNST	Suppress upstream RAS activation; synergy with TMZ *	NCT03114319

* Chemotherapy drug (alkylating agent).

**Table 2:** Current and ongoing clinical trial assessing certain MAPK inhibitors, as single agents or in combinations, for central nervous system (CNS) tumors. LGG, low-grade glioma; HGG, high-grade glioma; HQ, hydroxychloroquine; NF1, neurofibromin; PN, plexiform neurofibroma; OP/HG, optic pathway/hypothalamic glioma; PA, pilocytic astrocytoma.

Agent (Target)	Tumor Type	Age	Study Name/ Clinical Trial ID	Stage
Dabrafenib * and trametinib ^±^	*BRAF V600*-mutant pLGG	12 months–25 years old	NCT07110246	Phase II
Dabrafenib * and trametinib	Several CNS tumors	1–99 years old	NCT03975829	Phase IV
Dabrafenib, trametinib ^±^ and nivolumab ^⊥^	*BRAF*-altered pediatric glioma	1–26 years old	NCT06712875	Phase I/II
Dabrafenib * and trametinib ^±^	HGG (among other cancer types)	18–100 years old	NCT03340506	Phase IV
Dabrafenib * and trametinib ^±^	HGG	3–25 years old	NCT03919071	Phase II
Dabrafenib, trametinib ^±^ and HQ ^∝^	LGG or HGG with BRAF aberration LGG with NF1	1–30 years old	NCT04201457	Phase I/II
Mirdametinib ^±^	LGG	2–24 years old	NCT04923126	Phase I/II
Mirdametinib ^±^	LGG, activation of MAPK	2–24 years old	NCT06666348	Phase I/II
Selumetinib ^±^	Recurrent/refractory LGG, OP/HG glioma, NF1, PA	3–21 years old	NCT01089101	Phase I/II
Selumetinib ^±^	Progressive LGG	2–25 years old	NCT04576117	Phase III
Selumetinib ^±^	NF1, LGG	2–21 years old	NCT03871257	Phase III
Selumetinib ^±^	LGG	2–21 years old	NCT04166409	Phase III
Trametinib ^±^	LGG	1 month–25 years old	NCT05180825	Phase II
Trametinib ^±^	LGG, NF1, PN, activation of the MAPK/ERK pathway	1–25 years old	NCT03363217	Phase II
Trametinib ^±^ and everolimus ^∇^	LGG, HGG	1–25 years old	NCT04485559	Phase I
Tovorafenib *	Relapsed/refractory LGG with BRAF alterations	6 months–25 years old	FIREFLY-1/NCT04775485	Phase II

* RAF inhibitor, ^±^ MEK inhibitor, ^⊥^ anti-programmed cell death-1 (PD1) monoclonal antibody, ^∝^ autophagy inhibitor, ^∇^ mammalian target of rapamycin (mTOR) inhibitor.

## Data Availability

No new data were created or analyzed in this study.

## Abbreviations

The following abbreviations are used in this manuscript:

2-HG	2-hydroxyglutarate
ABC	ATP-binding cassette
AKT	protein kinase B (PKB)
ALK	anaplastic lymphoma kinase
ARAF	A-Raf proto-oncogene
ASIR	age-standardized incidence rate
ATRX	α-thalassemia intellectual disability X-linked
BBB	blood–brain barrier
BCAN	brevican
BCR	breakpoint cluster region
BCRP	breast cancer resistance protein
BRAF	v-Raf murine sarcoma viral oncogene homolog B
BTB	blood–tumor barrier
CAR	glioblastoma multiforme
CDK4	cyclin-dependent kinase 4
CDKN2A/B	cyclin-dependent kinase inhibitor 2A/2B
CLCN6	CLCN6 chloride voltage-gated channel
CLIP2	CAP-Gly domain containing linker protein 2
CNS	central nervous system
CRAF	proto-oncogene c-Raf
DHGG	diffuse high-grade glioma
DIG	desmoplastic infantile ganglioglioma
DLGG	diffuse low-grade glioma
EGFR	epidermal growth factor receptor
ERK	extracellular signal-regulated kinase
ETV6	Ets variant transcription factor 6
FAM131B	family with sequence similarity 131 member B
FDA	food and drug administration
FGFR	fibroblast growth factor receptor
FZR1	fizzy and cell division cycle 20 related 1
GBD	global burden of disease
GBM	glioblastoma multiforme
GNA11	guanine nucleotide-binding protein subunit alpha 11
GOPC	Golgi-associated PDZ and coiled-coil motif-containing protein
GTF2I	general transcription factor II-I
GTPase	guanosine triphosphatase
HGG	high-grade glioma
HQ	hydroxychloroquine
HRAS	Harvey rat sarcoma viral oncogene homolog
ICI	immune checkpoint inhibitor
IDH	isocitrate dehydrogenase
IHG	infant-type hemispheric glioma
IL-13Rα2	interleukine-13Rα2
KLF4	Krüppel-like factor 4
KRAS	Kirsten rat sarcoma viral oncogene homolog
LGG	low-grade glioma
LZTR1	leucine zipper-like transcription regulator 1
MACF1	microtubule actin crosslinking factor 1
MAPK	mitogen-activated protein kinase
MEK	mitogen-activated protein kinase extracellular signal-regulated kinase
MET	met tyrosine-protein kinase
MKRN1	makorin ring finger protein 1
MPNST	malignant peripheral nerve sheath tumor
MRP	multidrug resistance-associated protein
MYB	v-myb avian myeloblastosis viral oncogene homolog
NACC2	nucleus Accumbens-associated protein 2
NF1	neurofibromin
NF1A	nuclear transcription factor 1A (NF1A)
NFASC	neurofascin
NOTCH	neurogenic locus notch homolog
NRAS	neuroblastoma rat sarcoma viral oncogene homolog
NTRK	neurotrophic tyrosine receptor kinase
OLIG2	oligodendrocyte transcription factor 2
PA	pilocytic astrocytoma
PD-1	programmed cell death-1
PDGFR-A	platelet-derived growth factor receptor A
PD-L1	programmed death-ligand 1
PFS	progression-free survival
P-gp	p-glycoprotein
PI3K	phosphoinositide 3 kinase
PIK3CA	phosphatidylinositol-4,5-bisphosphate 3-kinase catalytic subunit alpha
PN	plexiform neurofibroma
PRKAR2A	protein kinase cAMP-dependent type II regulatory subunit α
PROTAC	proteolysis-targeting chimera
PTPRZ1	protein tyrosine phosphatase receptor type Z1
PXA	pleomorphic xanthoastrocytoma
QKI	quaking homolog KH domain containing RNA binding
RAF	rapidly accelerated fibrosarcoma
RAS	rat sarcoma virus oncogene
RB1	retinoblastoma susceptibility gene
RNF130	ring finger protein 130
ROS1	ROS proto-oncogene 1
RTK	receptor tyrosine kinase
SASP	senescence-associated secretory phenotype
SHP2	Src homology region 2 domain-containing phosphatase-2
SOX2	SRY-box transcription factor 2
SPRED1	sprouty-related EVH1 domain-containing protein 1
SRGAP3	rho GTPase activating protein 3
STAT3	signal transducer and activator of transcription 3
TACC3	transforming acidic coiled-coil containing protein 3
TERT	telomerase reverse transcriptase
TME	tumor microenvironment
TMZ	temozolomide
TPM3	tropomyosin 3
TrkB	tropomyosin receptor kinase B
WHO	world health organization
